# Hepatic artery embolization cures the acute pancreatitis associated with a tiny arteriobiliary fistula after TIPS: A case report

**DOI:** 10.1097/MD.0000000000009267

**Published:** 2017-12-15

**Authors:** Xiuli Yin, Xiaofei Lei, Changqing Xu, Jing Yang, Yingying Zhao, Kun Li

**Affiliations:** a Department of Gastroenterology, Shandong Provincial Rongjun Hospital; b Department of Gastroenterology, Shandong Provincial Qianfoshan Hospital of Shandong University, Jinan, Shandong Province, China.

**Keywords:** acute pancreatitis, arteriobiliary fistula, bile duct injury, transjugular intrahepatic portosystemic shunt (TIPS)

## Abstract

**Rationale:**

Esophageal variceal bleeding caused by portal hypertension is massive and life-threatening to those patients with decompensated liver cirrhosis. A transjugular intrahepatic portosystemic shunt (TIPS) can effectively stop bleeding. But the process of puncture may lead to bile duct injury and even form fistulas between the hepatic artery and bile duct.

**Patient concerns:**

The case report illustrated a 52-year-old Chinese female patient who underwent TIPS.

**Diagnoses:**

She suffered from acute upper gastrointestinal hemorrhage and acute pancreatitis because of the bile duct injury after TIPS.

**Interventions:**

The fistulas between the hepatic artery and bile duct was embolized.

**Outcomes:**

The acute upper gastrointestinal hemorrhage and acute pancreatitis of the patient were cured.

**Lessons:**

The arteriobiliary fistula should be paid more attention after TIPS while early-stage prevention should be carried out.

## 1 Introduction

The transjugular intrahepatic portosystemic shunt (TIPS) procedure is an invasive procedure that carries the risk of several complications such as massive bleeding,^[1,2]^ pseudoaneurysm formation caused by hepatic artery injury,^[3]^ arterial occlusion, arterioportal fistula, or arteriobiliary fistula formation.^[4]^ Once an arteriobiliary fistula is formed, blood may flow from the high-pressure hepatic artery to the low-pressure bile duct. This will cause upper gastrointestinal bleeding, bile duct obstruction, and even acute pancreatitis. Therefore, TIPS is as a therapeutic option in patients with decompensated portal hypertension and as second-line treatment for variceal bleeding when endoscopical and pharmacological treatment fail. However, no case of acute pancreatitis associated with an arteriobiliary fistula after TIPS have been reported. This report describes a case of acute pancreatitis resulting from the bile duct and the hepatic artery fistula after TIPS. The patient was cured after the arteriobiliary fistula was embolized. Written consents were obtained from the patient according to the ethical guidelines of our institution. This case report was exempt from approval by the medical ethics committee at our hospital.

## 2 Patient

**Figure 1:**
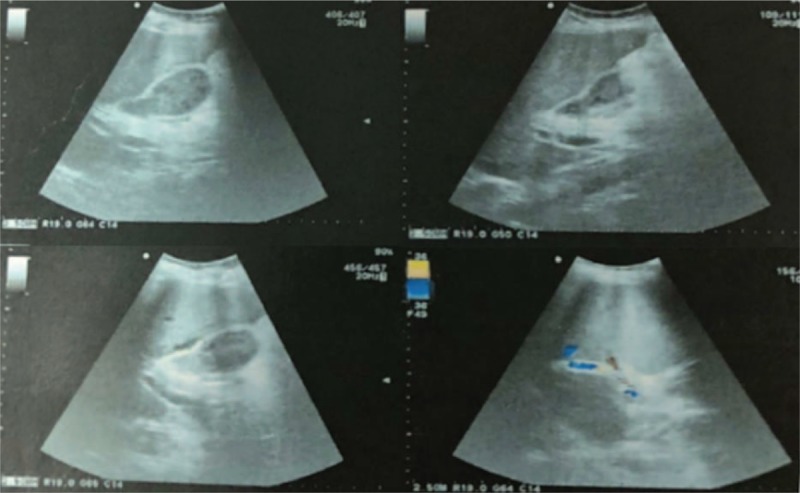
Figures of ultrasound after TIPS. There is moderately strong echo filling in the gallbladder and CBD. CBD = common bile duct, TIPS = transjugular intrahepatic portosystemic shunt.

**Figure 2:**
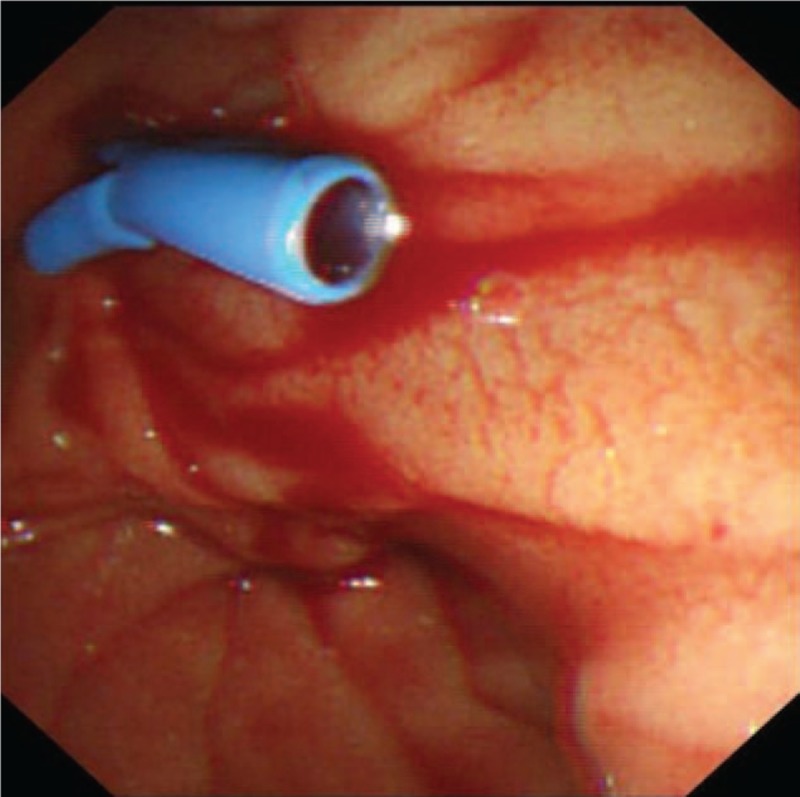
A plastic stent was placed in the CBD by ERCP. It can be seen that bloody fluid drained out of the blue catheter. CBD = common bile duct, ERCP = endoscopic retrograde cholangiopancreatography.

**Figure 3:**
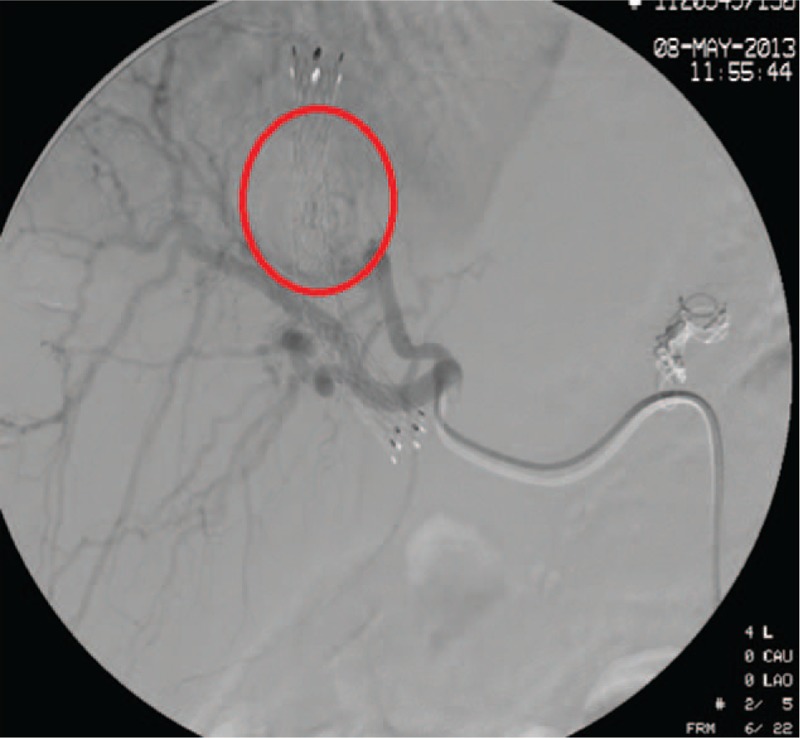
Hepatic arteriography after the embolization showed that no filling of hepatic arterial branches adjacent to the TIPS stent (the red circle region). TIPS = transjugular intrahepatic portosystemic shunt.

A 52-year-old Chinese female, with recurrent gastrointestinal bleeding due to primary biliary cirrhosis, had undergone the TIPS procedure in our hospital. Her model for end-stage liver disease score was 6, white blood cell count 2.01×10^9^/L, red blood cell (RBC) count 3.83×10^12^/L, and hemoglobin 10.9 g/dL before the operation. The amylase, lipase, and pancreatic amylase were within normal ranges before TIPS. The abdomen ultrasound examination showed no abnormality in the bile duct. The Rösch–Uchida Transjugular liver accessory set we used was RUPS-100 (Cook, USA). TIPS was created with a polytetrafluoroethylene-covered stent (Viattor, Gore) between the right hepatic vein and right portal vein. The mean hepatic venous pressure gradient dropped from 27 to 7 mm Hg. The puncture process from the hepatic vein to portal vein was repeated 5 times. One day after TIPS, the patient felt severe abdominal pain, and defecated tarry stool 3 times. Her RBC decreased to 3.12×10^12^/L, hemoglobin decreased to 6.2 g/dL, amylase 582 IU/mL, lipase 1332 IU/mL, pancreatic amylase 537 IU/mL, and a fecal occult blood test was positive. Ultrasound examination showed moderately strong echo filling in the gallbladder and common bile duct (CBD) (Fig. 1). The patient was diagnosed with acute upper gastrointestinal bleeding and acute pancreatitis. A plastic stent was placed in the CBD by endoscopic retrograde cholangiopancreatography (ERCP), and bloody fluid was seen in the stent (Fig. 2). One day after ERCP, the symptom of abdominal pain disappeared, after 2 days, the amylase decreased to 49 IU/mL, the lipase decreased 69.2 IU/mL, and the pancreatic amylase decreased to 48 IU/mL. Ultrasound examination showed that the echogenic structure in the CBD had disappeared. But the RBC and the hemoglobin continued to decrease. We suspected injuries in the puncture process, resulting in a fistula between the bile duct and hepatic artery. The patient underwent hepatic arteriography, but no arteriobiliary fistula was found. We considered that the fistula might be too small to be found, so we tried to embolized the tiny branches of the hepatic artery with gelatin sponge particles in the puncture region. Hepatic arteriography was performed again after the embolization, and the hepatic artery branches around the stent disappeared (Fig. 3). Three days after hepatic arterial embolization, the color of her stool became yellow, and the stent in the CBD was removed. Six days later, the patient was discharged from our hospital without any complication. The patient was followed up to July 2016, with no recurrence of gastrointestinal bleeding and no abdominal pain.

## 3 Discussion

The first successful TIPS was done by Rössle, Richter, Nöldge, and Palmaz at the University of Freiburg in 1988.^[5]^ With the development of technology and experience, technical success rate of the procedure was >95% with a major complication rate of <5% in the 90s of the last century.^[6,7]^ However, the creation of TIPS is still a huge challenging procedure performed by interventional radiologists. The complications of TIPS including hemorrhage, encephalopathy, TIPS dysfunction, and liver failure are well known. But the complications associated with the process of liver puncture are closely related to the skill of the operators, and as the consequences are serious, they must be anticipated in order that they can be diagnosed and treated in an expeditious manner. Although puncture of the biliary duct is common during TIPS, severe bile duct injury is reported to be present in <1% of cases.^[8]^ Compared with the biliary–venous fistula, the arteriobiliary fistula is rarer and the consequence is more serious. The arteriobiliary fistula might be formed by repeated punctures during the process of TIPS, and it connects the hepatic artery and bile tree, which leads blood from the high-pressure hepatic artery flows to the low-pressure bile duct, and causes the formation of blood clots, blocking the biliary system, eventually leading to acute pancreatitis. In this report, the puncturing process of the portal vein before TIPS implantation was repeated 5 times and the quite high number of approaches are probably the reason for development of the arteriobiliary fistula. However, 3-dimensional CT before operation can fully show hepatic and portal venous anatomy for TIPS, which will decrease the complications of TIPS. Once the arteriobiliary fistula is verified by hepatic arteriography, it can be cured with embolization of the catheter track or the bleeding branch of the hepatic artery,^[9,10]^ or placement of a covered stent in the biliary system.^[11]^ If the arteriobiliary fistula is tiny, as our report, it cannot be found by the arteriography. We found hemobilia by duodenoscope during the process of ERCP which helped to confirm the presence of a tiny arteriobiliary fistula. The pressure of the hepatic vein was low, and the pressure of the portal vein declined after TIPS, so we concluded that the hemobilia was due to the arteriobiliary fistula rather than a biliary–venous fistula. In our patient, considering the advanced nature of the disease, placement of a plastic stent through ERCP was thought to be reasonable as a first attempt because it helped to cure the acute pancreatitis, but the hemobilia continued. So, we tried to embolize these repeated puncture regions where a tiny fistula might exist with gelatin sponge particles. The clinical course confirmed that our approaches were effective. Of course, we must pay attention to avoid too large embolized area, otherwise it may lead to liver necrosis or acute liver failure. The acute pancreatitis associated with a tiny arteriobiliary fistula after TIPS must come to the operator's attention, must be detected early, diagnosed, and timely treatment given, otherwise it may lead to severe consequences.

## Acknowledgment

The authors thank Dr Edward C Mignot, Shandong University, for linguistic advice.
